# Epitope Expression Persists in Circulating Tumor Cells as Breast Cancers Acquire Resistance to Antibody Drug Conjugates

**DOI:** 10.1101/2025.04.02.646822

**Published:** 2025-04-03

**Authors:** Avanish Mishra, Rachel Abelman, Quinn Cunneely, Victor Putaturo, Akansha A. Deshpande, Remy Bell, Elizabeth M. Seider, Katherine H. Xu, Mythreayi Shan, Justin Kelly, Shih-Bo Huang, Kaustav A. Gopinathan, Kruthika Kikkeri, Jon F. Edd, John Walsh, Charles S. Dai, Leif Ellisen, David T. Ting, Linda Nieman, Mehmet Toner, Aditya Bardia, Daniel A. Haber, Shyamala Maheswaran

## Abstract

Antibody-drug conjugates (ADCs) targeting cell surface proteins TROP2 or HER2 are effective in metastatic breast cancer, but the precise clinical contribution of epitope expression is uncertain. We prospectively monitored circulating tumor cells (CTCs) in 33 patients receiving ADC therapies using quantitative imaging. The expression of TROP2 and HER2 are heterogeneous across single CTCs from untreated patients, comparable to matched tumor biopsies, and display poor association with clinical response. Within three weeks of treatment initiation, declining CTC numbers correlate with a durable response (TROP2: median time to progression 391 versus 97 days, HR 4.15, P=0.0046; HER2: 322 versus 66 days, HR 9.12, P=0.0002). Neither TROP2 nor HER2 expression is reduced at progression, compared to matched pretreatment CTCs, and switching ADC epitope while maintaining a similar payload shows poor efficacy. Thus, epitope downregulation is not a common driver of acquired resistance to TROP2 or HER2 ADCs, and second-line ADC therapies may benefit from distinct payloads.

**SIGNIFICANCE:**

ADCs target tumor-associated antigens, followed by internalization and release of drug payloads. However, clinical studies of epithelial-targeting ADCs show efficacy despite low tumor epitope expression. Our finding that epitope downregulation does not commonly accompany acquired resistance suggests alternative drivers of clinical efficacy and the need for testing non-cross-resistant payloads to overcome resistance.

## INTRODUCTION

ADCs combine an antibody targeting a tumor cell-enriched epitope with a cytotoxic payload, thereby providing the potential to deploy more selective chemotherapy with a favorable therapeutic window and toxicity profile (1). This specific delivery of the payload to cells containing tumor-associated antigens allows the use of drugs substantially more toxic than traditional chemotherapy (2). ADCs were first deployed in breast cancer for the treatment of metastatic breast cancer (MBC) with *HER2* gene amplification (so-called HER2-positive subsets) (3). More recently, ADCs have been FDA-approved in MBCs lacking *HER2* gene amplification, including two that target the cell surface epitope TROP2 (sacituzumab govitecan [SG] and datopotamab deruxtecan [Dato-DXd]), and one targeting HER2 (trastuzumab deruxtecan (T-DXd)) (4–6). Given the success of these ADCs, there are many more at various stages of development for breast cancer, as well as other solid tumors (6–9). Of note, the three FDA-approved ADCs contain payloads with highly potent, albeit functionally related, topoisomerase-I (TOP1) inhibitors. While ADCs have proven to be highly effective in many patients with advanced breast cancer (10), other patients exhibit *de novo* resistance or acquire drug resistance after only a few months of therapy. Retrospective institutional series have suggested that MBC patients who receive a first-line ADC often experience shorter times on treatment with second-line ADCs (11–13). Therefore, biomarkers are needed to optimize the selection and sequencing of existing ADCs and to guide the development of next-generation agents (14).

Efforts to use tissue-based antigen expression to guide ADC selection have produced mixed results. TROP2, the target of SG and Dato-DXd, is more highly expressed in normal developing epithelial tissues compared with adult organs, and it is aberrantly expressed in certain tumors, including both triple-negative breast cancer (TNBC) and hormone receptor (HR)-positive breast cancer (15–18). Biomarker studies of SG have suggested a poor correlation between clinical response and the degree of TROP2 expression, as measured in tumor biopsy specimens, either at the time of initial disease presentation or subsequent metastatic relapse (19–21). A “bystander effect,” whereby ADCs recruited to TROP2-expressing cells may then kill surrounding cells lacking the antigen, has been postulated (22). However, archival specimens obtained from a single metastatic lesion may not fully address the potential spatial and temporal heterogeneity of TROP2 expression in cases of progressing MBC. Similarly, ADCs targeting HER2, such as T-DXd, have demonstrated efficacy in HER2-positive cancers lacking *HER2* gene amplification and its associated biological dependence on HER2 signaling. This efficacy extends to HER2-low breast cancers (median PFS of 9.9 months for T-DXd vs. 5.1 months in physician’s choice chemotherapy, HR 0.5, $p<0.001$) (6). Most recently, breast cancers with virtually no detectable HER2 expression were shown to be as responsive to HER2-ADCs as those with low levels of expression, leading to the expansion of FDA approval while also raising questions about the tumor epitope dependence of HER2 ADC therapy (23,24).

Non-invasive liquid biopsies analyzing circulating tumor cells (CTCs) may enable serial analysis of protein expression by intact cancer cells drawn from blood samples of patients receiving ADC therapies. Whereas primary tumor biopsies taken at initial diagnosis do not reflect dynamic changes induced by cancer treatment and progression, metastatic tumor biopsies are typically driven by anatomy, and they sample a single site of disease that may not capture the heterogeneity of advanced cancer. In contrast, CTCs are shed into the bloodstream from all tumor deposits, thereby representing the patient's entire tumor burden, and immunofluorescent staining of single CTCs can enable quantitative analyses of tumor cell heterogeneity (25–28). Importantly, the technology employed for CTC enrichment from blood samples may influence the measurement of cell surface epitopes: the capture of CTCs based on their expression of epithelial cell surface markers, such as EpCAM, may lead to selection bias for co-expressed epithelial markers, while CTC enrichment based on tumor cell size fails to detect the approximately 65% of CTCs whose diameter overlaps with that of leukocytes (28). Here, we used a microfluidic technology that effectively depletes red blood cells and platelets using fluidic wall lift forces and removes leukocytes using magnetically conjugated antibodies, thus enriching CTCs without bias for either size or epithelial differentiation (29–33). Among CTCs isolated, the application of quantitative multispectral imaging for the ADC target epitopes of interest enables single-cell analysis of tumor cell heterogeneity (**Figure 1**). We applied this technology for serial monitoring in 33 patients (36 treatment courses) with breast cancer treated with either TROP2 or HER2 targeting ADCs or sequentially with two ADCs.

## RESULTS

### Quantitative imaging of TROP2 and HER2 epitope expression in CTCs using multispectral imaging

We have previously described a microfluidic leukocyte-depletion platform (CTC-iChip) that excludes non-nucleated cells and then achieves efficient removal of magnetically tagged WBCs using antibodies against the common leukocyte epitopes CD45, CD16, CD66b, thereby providing unbiased enrichment for CTCs (30,31) (**Figure 1**). The enriched CTCs themselves are free of conjugated antibodies and magnetic beads, providing optimal resolution for quantitative imaging. While the CTC-iChip platform previously required immediate processing of freshly collected blood specimens, we adapted the analytical technology to allow fixation of blood specimens at the time of collection, thus increasing the time available for specimen processing up to 72 hours (**Supplementary Figure 1**). This was accomplished by collecting blood in Streck tubes that enable uniform light fixation and by imaging cells using a multispectral microscopy platform with subcellular resolution (PhenoImagerHT, Akoya Biosciences). Applying this revised processing approach, we also observed that reducing the concentration of leukocyte-tagging antibodies and magnetic particles by up to four-fold results in high CTC enrichment (96.9±5.1%), with only a modest reduction in CTC purity and without compromising single-cell CTC imaging and enumeration (**Supplementary Figure 1B**). Modeling spiking of cultured CTCs into Streck-fixed blood samples stored for 24 hours shows a CTC recovery of 96.9±5.1%, a $Log_{10}$ WBC depletion of 2.88±0.56, and a $Log_{10}$ RBC depletion of 4.19±0.47. A schematic representation of the CTC analytic platform is shown in **Figure 1**.

CTCs are classically defined as cells in circulation that are positive for epithelial markers and negative for leukocyte markers (34). To quantitatively measure the expression of ADC-targeted epitopes across all detectable CTCs, we applied high-resolution (40X) multispectral imaging, taking advantage of sharp emission spectra that allow for scoring multiple epitopes with minimal cross-fluorescence (35). Breast CTCs were identified as intact, nucleated, DAPI-positive cells that also stain positive for one or all of the Epithelial Cell Adhesion Molecule (EpCAM), pan-cytokeratin (CK), or cytokeratin 19 (CK19) markers (all grouped in the Alexa Fluor 488). Cells were simultaneously stained using antibodies against TROP2 (Alexa Fluor 555) and/or HER2 (Alexa Fluor 594) to score for CTCs expressing these ADC targets (**Supplementary Table 1**). Commonly used standard breast cancer cell lines with high, medium, or low expression of TROP2 or HER2 were used to properly scale the quantification of these markers in CTCs (**Supplementary Figure 2**). Digital image segmentation analysis was used to identify initial CTC images, which were then confirmed manually. Representative images of EpCAM/CK-positive CTCs expressing TROP2 and HER2 are shown in **Figure 2A**. Among all patients with metastatic breast cancer studied, 29 of 36 (81%) cases had ≥3 CTCs at baseline of sufficient quality for imaging analysis within 20mL of whole blood. Among these cases, the mean number of CTCs was 174.1 (8.70 CTCs/mL whole blood) (4.0-1481 CTCs), with a median of 59.5 (2.97 CTCs/mL).

**Comparison of TROP2 and HER2 protein expression in matched tissue biopsies and CTCs**

As a first step toward deploying CTC imaging as a non-invasive platform for ADC epitope scoring, we compared CTCs with patient-matched biopsies of metastatic tumors. Of the seven patient samples, four were obtained prior to any ADC therapy. When possible, matched CTC samples were collected within 3 months of the clinically mandated biopsy. Traditional immunohistochemistry (IHC) staining is poorly quantitative and may be further complicated in bone metastases, which are common in breast cancer and require tissue decalcification. We therefore deployed multispectral fluorescence-based imaging at the single-cell level for both CTCs and matched tumor biopsies using the same fluorescent-conjugated antibody cocktails and identical single-cell algorithms (**Figure 2A-B**; see Methods).

In metastatic tumor biopsies, TROP2 levels span a wide range of expression, which we annotated for scoring purposes using breast cancer cell lines: MDA-MB231 (low), AU565 (medium), and BRx142 (high), while the melanoma CTC cell line Mel167 was used as a negative control to establish null cut-offs (**Supplementary Figure 2**). Cancer cells (EpCAM, pan-cytokeratin, and CK-19 positive) within tumor biopsies and CTCs were scored at the single cell level for TROP2 expression. Tumor biopsies and CTC analyses were scored as concordant (+) or discordant (-) depending on the shared presence or absence of medium/high TROP2 expression. Across seven cases, four were concordant in having positive TROP2 scoring (MGH002, 039, 022, and 043). In the three discordant cases (MGH040, 041, and 042), positive staining of some cells in tumor biopsies was not captured by the relatively small number of isolated CTCs (**Figure 2C**).

HER2 staining of the same seven biopsies was calibrated by comparison with standard breast cancer cell lines: MDA-MB231 (low), BRx142 (medium), and AU565 (high), again with melanoma line Mel167 used as a negative control. Six of the seven cases showed concordance between tumor and CTC analyses, all scoring positive for HER2 expression (MGH002, 039, 022, 041, 042, and 043). In the discordant case (MGH040), positive HER2 expression by tumor cells was not evident in the small number of captured CTCs (**Figure 2D**). Taken all together, when analyzed at the single cell level with quantitative multispectral imaging, significant heterogeneity in both TROP2 and HER2 expression is evident in tumor biopsies, as well as in matched CTCs. Within the constraints of a small number of CTCs potentially representing all sites of metastatic disease, compared with the much larger number of cancer cells present in a single metastatic lesion, concordance between biopsies and CTCs was evident in ten of fourteen paired specimens (71%).

**TROP2 expression in serially collected CTCs from patients treated with TROP2-targeting ADC**

We prospectively studied 17 patients with metastatic breast cancer (**Supplementary Table 2**) treated with a TROP2-targeting ADC, analyzing the number of CTCs and their individual TROP2 expression levels immediately before initiation of therapy, at three weeks on treatment and at the time of disease recurrence. TROP2 expression was classified as low, medium, or high using the same calibration that we had applied in the different cases used for the concordance study (**Supplementary Figure 2**), and it was standardized across each analysis using the same reference breast cancer cell lines with different levels of TROP2. Among treated patients, we classified cases as having a poor response if they were maintained on therapy for less than 6 months (eight patients; 47%) or as having a strong response if they continued therapy beyond that time point (nine patients; 53%). Five of the nine strong responders stayed in therapy for over a year (**Figure 3A**). Among all 17 cases, CTCs were detectable at the pretreatment baseline in 13 cases (76.5%, mean number: 85±117 CTCs/20mL, median: 34 CTCs/20mL). The four cases with no detectable CTCs at baseline all had strong responses to ADC therapy. As expected, the quantitative expression of TROP2 in the 13 cases with ≥3 CTCs was heterogeneous. Three of the four (75%) cases in which at least 5% of CTC had medium or high TROP2 expression subsequently had a strong response compared with two out of the nine (22%) who had fewer than 5% CTCs. While not reaching statistical significance due to a small cohort size, these results lend some support to the concept that a small fraction of cancer cells expressing TROP2 may recruit the ADC to the tumor, with additional tumor lysis resulting from a bystander effect (2).

In addition to baseline epitope expression, rapid response to ADC therapy may be clinically impactful in allowing treatment adjustments prior to the often-delayed radiographic evidence of treatment response. Of 15 patients who were tested for CTCs at both baseline and follow-up three weeks after the first dose of TROP2-targeting ADC, 11 cases had either no detectable CTCs or a greater than 80% decline in CTC number at this early post-treatment timepoint; 9 (82%) of these went on to have a strong response. In contrast, of four patients who either had a smaller on-treatment decline in CTC numbers or an increase in CTCs (MGH012), none (0%) had a strong response to TROP2-directed ADC (median 391 vs 97 days for TROP2, hazard ratio 4.15, p = 0.0046) (**Figure 3B**).

**Analysis of serially collected CTCs from patients receiving HER2-ADC**

We extended our analysis to another 13 patients receiving the HER2-targeting ADC T-DXd for HR-positive, HER2-negative (HR+/HER2-) MBC. Seven of 13 (54%) patients were classified as having a strong response (remaining on treatment for more than six months), whereas six patients (46%) had a poor response (**Figure 3C**). Within individual patients, the pretreatment baseline displayed considerable heterogeneity in HER2 expression by single CTCs, showing no clear correlation with the duration of response (**Figure 3C**). Consistent with previous studies, staining available tissue biopsies for HER2 expression using standard clinical IHC scoring parameters also did not correlate with ADC response (**Supplementary Figure 3**). However, as with TROP2-targeting ADCs, a profound immediate decline in CTC numbers three weeks after initiation of therapy was highly correlated with response (**Figure 3D**). Of eight patients with a greater than 80% decline in CTC numbers or no detectable CTCs at the earliest timepoint post-treatment initiation, seven (77.8%) had a strong response to ADC therapy, while none of three (0%) patients without such a CTC decline had a strong response (322 vs 66 days for HER2, hazard ratio 9.12, p = 0.0002) (**Figure 3D**).

## TROP2 or HER2 epitope expression at clinical progression on ADC therapy.

To test whether epitope downregulation contributes toward acquired clinical resistance to the targeting ADC, we compared CTCs at the time of drug discontinuation with the matched pretreatment baseline for both TROP2 and HER2-targeting ADCs (**Figure 4**). Remarkably, none of the nine cases for which paired specimens were available for analysis showed downregulation of TROP2 expression at the time of clinical progression on a TROP2-targeting ADC (**Figure 4A**): in eight patients (89%), the single cell distribution in TROP2 expression was comparable between baseline and resistance timepoints, while in one patient (11%) (MGH009) there was a striking increase in CTC TROP2 expression, despite disease progression after a prolonged 550-day response (**Figure 4B**). As shown in representative images, TROP2 expression and specific morphological characteristics, such as CTC clustering, persisted from the time of ADC sensitivity before therapy to drug-resistant recurrence (**Figure 4C**).

In seven patients with matched pretreatment and resistance samples following HER2-targeting ADC, five (71%) showed no meaningful change in HER2 expression at recurrence, while one (14%) had a significant increase in HER2 levels, and one (14%) had a decrease in HER2 (**Figure 4D-E**). Again, representative images illustrate the unchanged morphological appearance in HER2-expressing CTCs before treatment initiation and after discontinuation of therapy (**Figure 4F**). Thus, for both TROP2 and HER2, epitope expression persists in CTCs of most patients whose disease progressed on ADC therapy, suggesting that epitope downregulation is not a common cause of acquired resistance.

## CTC analysis in second-line ADC therapy

We analyzed CTCs enriched from two patients who had previously received a HER2-directed ADC and switched at the time of treatment failure to receive TROP2-ADC as a second-line therapy, and conversely from four patients receiving TROP2-ADC after an initial HER2-ADC (**Figure 5A-B**). In comparison to first-line ADC cases, all six patients had poor responses to the second-line ADC (median time to progression: 229 days for any first-line ADC therapy versus 90 days for any second-line therapy), irrespective of baseline epitope expression or on-treatment changes in CTCs (**Figure 5C**). Of note, both HER2- and TROP2-directed ADCs used in these cases carry camptothecin-related drug payloads, again supporting the notion that treatment failure may be driven by acquired resistance to the shared payload rather than downregulation of the distinct targeted epitopes. Both TROP2 and HER2 are cell surface epitopes that are linked to cellular lineage and differentiation status, and while they are overexpressed in some cancer cells, they are not tumor-specific and are present in multiple normal tissues. Given the frequency with which ADCs targeting TROP2 or HER2 are considered for sequential clinical applications, we interrogated single CTCs and single cells from tumor biopsies for coexpression of these two epitopes. Among the seven cases stained for both TROP2 and HER2 as part of our initial CTC vs tumor biopsy concordance analysis (Figure 2), we observed correlations in the expression of these two epitopes within single breast cancer CTCs ($r = 0.49$, $P < 0.0001$) and within single tumor cells from metastatic biopsies ($r = 0.68$, $P < 0.0001$) **(Figure 5 D and E)**. Taken together, TROP2 and HER2 represent two epithelial cell surface markers that are relatively enriched in some breast cancer cells, with overlapping baseline expression patterns, which are not considerably altered in most cases that acquire resistance to ADC therapy.

## DISCUSSION

We describe the application of quantitative single-cell CTC imaging analysis for serial expression of tumor epitopes targeted by ADC therapies in breast cancer. Currently approved indications for ADCs in breast cancer are directed at tumors that are refractory to first-line therapies and, hence, may have undergone selective evolutionary pressures affecting their expression of epithelial, mesenchymal, and other lineage-related markers. The relative ease of blood-based sampling, together with technical improvements in the stabilization of blood specimens and increased CTC yields from the interrogation of larger (20 mL) blood volumes with fewer WBC-depleting beads, make CTC analyses a realistic alternative to invasive biopsy of metastatic tumor deposits. As such, CTC analyses enable longitudinal, blood-based, non-invasive monitoring of protein expression on cancer cells as they initially respond and ultimately progress on ADC therapies.

While expression of either TROP2 or HER2 on single CTCs from individual patients shows a high degree of heterogeneity, we note a general concordance between measurements based on CTCs and metastatic tumor biopsies. The relatively few discordant results observed here include cases in which only a small number of CTCs were detected, thereby inadequately sampling the tumor burden. In the future, serial sampling or larger blood volumes could overcome the limitations posed by a few CTCs. It is also possible that in other discordant cases, the single lesion biopsied may not be as representative as CTCs of all metastatic lesions in the patient.

Consistent with previously documented biopsy studies (20,21), baseline expression of neither TROP2 nor HER2 is highly predictive of ADC response in breast cancer. Tumor cell heterogeneity in both TROP2 and HER2, evident in both tumor biopsies and CTCs, may partially explain the poor predictive value of baseline epitope expression. In the case of SG, the linker connecting the drug payload to the antibody is highly susceptible to spontaneous hydrolysis (2), and recruitment of the ADC by a small number of high TROP2-expressing cancer cells may lead to its release in the tumor microenvironment and killing of bystander cancer cells with low TROP2 expression (23,24). In our small cohort, we observed a trend toward strong responses in patients who had at least 5% CTCs with medium or high TROP2 expression. However, no such correlation was observed for HER2-targeting ADC.

Our study thus illustrates an emerging paradox in antibody-directed therapies for cancer. ADCs enable the administration of highly toxic payloads that would not be tolerated without conjugation to an antibody, but the selective internalization of the drug within tumor cells does not solely reflect antibody-dependent targeting of tumor cells. In cases with highly differential expressions of an epitope, such as HER2-amplified breast cancer or rare lineage epitopes that are uniquely expressed on tumor cells, the rationale for ADC selectivity based on tumor cell expression is compelling. However, for more common epitopes expressed on many epithelial cells, with less differentially increased expression on cancer cells, other factors may contribute. These include pharmacokinetic parameters of ADC distribution, with accumulation at sites of vascular permeability or even slow release from normal tissue reservoirs (23). Alternatively, as proposed for SG, it is possible that a bystander effect might lead to clinical efficacy driven by a small number of epitope-expressing tumor cells within the tumor microenvironment. However, in contrast to HER2 and TROP2 ADCs, multiple other ADCs with comparable differential epitope expression profiles have not achieved their expected clinical endpoints (36). Thus, effective development and deployment of ADCs for the treatment of cancer will require a comprehensive understanding of pharmacokinetics parameters, payload sensitivity profiles, and tumor epitope biomarkers to predict clinical response.

For both TROP2 and HER2-targeting ADCs, we find the greatest potential clinical value for CTC analyses in the rapid measurement of tumor response. Indeed, three weeks after a single administration of either ADC, we find that the persistence of CTCs is correlated with a relatively short response, whereas a substantial decline or total absence of CTCs is correlated with a favorable outcome. Such rapid and quantitative assessment of response is not readily achievable using standard clinical radiographic assessments, which are typically delayed, and the calculation of changes in overall tumor burden using ctDNA measurements is complicated by the need to integrate diverse mutations, each with variable allele frequencies. Given the poor performance of sequential ADC administration, choosing the best ADC for first-line treatment is critical, and an immediate marker of tumor response may identify patients who could benefit from an early switch in ADC therapy. Additional studies will be required to test the clinical utility of early on-treatment CTC enumeration in ADC selection.

The most unexpected result from our use of prospective serial CTC measurements in the setting of ADC therapies is the absence of epitope downregulation at the time of acquired clinical resistance. Remarkably, we found that almost all ADC-resistant cases display comparable TROP2 and HER2 expression patterns as their matched pretreatment analyses, with a few cases even showing increased epitope expression at the time of acquired ADC resistance. This strongly suggests that for TROP2 and HER2 ADCs, tumor cell epitope expression is neither the driver of initial sensitivity nor that of acquired resistance. Acquired resistance to ADCs may be multifactorial and include a significant fraction of mutations associated with resistance to the camptothecin drug payload (14), as well as less well-understood alterations in drug import and metabolism (22,37). Our observations that the downregulation of epitopes by tumor cells is not a common driver of clinical failure for these ADCs should be considered together with the fact that switching targeting epitope between TROP2 and HER2, while preserving the camptothecin-related payload, leads to poor second-line responses. Thus, the drug payload, rather than the targeting antibody, may drive acquired resistance in TROP2 and HER2-directed ADCs, and prospective trials will need to evaluate whether changing the conjugated chemotherapeutic agent in second-line therapies may lead to effective second-line responses.

## Materials and Methods

### Sample Collection – ADC Cohort

Peripheral blood samples from patients were collected into 1 to 2 vacutainers—either into standard EDTA or Streck tubes—according to protocols reviewed and authorized by the MGB Institutional Review Board (DF-HCC protocol 13-416). Informed consent was obtained from all study participants. All human subjects in this study are females. For the ADC cohort, the inclusion criterion consisted of adult metastatic breast cancer patients starting TROP2-ADC or HER2-ADC therapy. The timepoints of these draws varied between patients due to differing clinical courses but generally coincided with the start of the first two cycles of treatment, radiographic staging, and regular follow-ups or recurrences. All patients tolerated the venipuncture well. Whole blood donations from healthy donors within our research group were collected in accordance with MGB IRB Protocol # 2009-P-000295. Additional whole blood samples from healthy donors were procured from Research Blood Components, LLC (Brighton, MA).

### CTC-iChip Fabrication and Sample Processing

Upon receipt, sample volumes were measured, incubated sequentially with antibodies and beads, and processed through the CTC-iChip. The CTC-iChips used to process samples were fabricated with medical-grade cyclic olefin copolymer (COC) using variotherm injection molding by Stratec Biomedical AG (Germany). Briefly, the devices utilize a pressure-driven flow of whole blood (diluted 1:1 with buffer) containing magnetically labeled WBCs and buffer to enrich CTCs. As the sample passes through the chip, it first flows through 16 inertial separation array devices, which remove the majority of platelets, RBCs, and unbound magnetic beads on the basis of size, allowing CTCs and WBCs to pass. Subsequently, cells are focused into a single-cell stream, and two stages of magnetic depletion channels use magnets outside the chip to separate labeled WBCs from CTCs and unlabeled cells. The final product, concentrated to a fraction of the initial sample volume, is collected while waste fractions are discarded.

Each product containing residual white and red blood cells and any enriched CTCs is kept on ice to preserve cellular morphology. To ensure consistency in downstream work, nucleated cells were enumerated for each sample and split if needed to plate ~100,000-300,000 cells on each slide. Cells were incubated in 0.5% paraformaldehyde for 10 minutes and cytospun using Epredia™ EZ Megafunnels and a Thermo Shandon Cytospin 4 instrument (2000 rpm, 5 minutes). In lieu of the included Megafunnel slides, TruBond™ 380 slides were used. Following the cytospin, slides were washed once with 1X PBS and air-dried to ensure maximal cell retention. Air-dried slides were stored for 1-2 nights at 4C until staining.

**WBC depletion antibody preparation**

A cocktail of antibodies was utilized to deplete WBCs in samples. These included biotinylated anti-human CD45 (Thermo Fisher Scientific, clone HI30, IgG1, 0.189 µg/million cells), biotinylated anti-human CD16 (BD Biosciences, clone 3G8, IgG1, 0.0189 µg/million cells), and biotinylated anti-human CD66b (Novus Biologicals, clone 80H3, IgG1, 0.0189 µg/million cells). To prepare the cocktail with the necessary concentrations, the three antibodies were diluted into sterile, filtered 1X phosphate-buffered saline (PBS) containing 0.1% bovine serum albumin (BSA) (Sigma-Aldrich #A2058) to improve stability. The cocktail was stored at 4ºC and used within 7 days.

**Magnetic beads preparation**

1 µm Dynabeads MyOne Streptavidin T1 superparamagnetic beads from Invitrogen were used to magnetically label WBCs with biotinylated antibodies. Before use, beads were washed with three washes of 0.01% Tween-20 (Fisher Scientific BP337-100) in 1X PBS to remove the storage solution, followed by three washes with 0.1% BSA in 1X PBS. Subsequently, beads were stored in the same 0.1% BSA solution at a concentration of 10mg/mL, 10,000,000 beads/uL to minimize nonspecific binding. As with the depletion antibody cocktail, washed beads were stored at 4ºC and used within 7 days.

**Buffer preparation**

The running buffer for the CTC-iChip, also used for washing and diluting samples and products, was composed of 0.2% Pluronic F-68 (Sigma-Aldrich #K4894) diluted in 1X PBS. Briefly, the polymer was dissolved in PBS until visually dispersed before filtering with a rapid flow 0.22µm filter (Corning #430517). The running buffer was stored at room temperature and used within 2 weeks.

**Staining**

For CTC staining, cells were blocked with a solution of 3% BSA and 2% Normal Goat Serum (Abcam #ab7481) in PBS for 1 hour and stained with EpCAM, panCK (CK8/18), and CK19 conjugated to Alexa Fluor� 488 (Cell Signaling Technology 5198S, Cell Signaling Technology 4523S, Thermo Fisher Scientific MA5-18158), pooled white blood cell markers CD45, CD16, and CD66b conjugated to Alexa Fluor� 647 (BioLegend 302020, BioLegend 304020, BioLegend 305110), TROP2 conjugated to Dylite-550 (Novus Biologicals NBP2-89492R, NBP2-89493R) or TROP2 labeled with Alexa Fluor� 555 (abcam ab214488, abcam ab150078), and HER2 labeled with Alexa Fluor� 594 (abcam ab11710, abcam ab150160), and DAPI (ThermoFisher Scientific 62248). Staining for all markers was done for 1 hour, using a solution of 0.1% BSA and 1% Tween-20 in PBS as diluent. Conjugated antibodies, DAPI, and any unconjugated primary antibodies were applied first. After two 5-minute washes, secondary antibodies were applied. Final washing was performed sequentially with 0.3% Tween-20 in PBS and 1X PBS before applying mounting media (Thermo-Scientific #P36962), a coverslip, and curing overnight. For the concordance samples, tissue biopsies were preserved in an FFPE block and cut into 5 µm thick sections. Normal human placenta FFPE slides were used as run controls. Prior to staining, FFPE slides were baked upright in an oven at 65°C for 3 hours. Both concordance CTC and FFPE tissue slides were stained using a Leica Bond RX automated stainer. Prior to blocking, heat-mediated antigen retrieval was performed with Bond ER2 solution (Tris-EDTA buffer pH 9.0) for 40 mins on the FFPE slides. All incubations were done at room temperature. Slides were coverslipped using ProLong Gold Antifade Mountant with DAPI.

**CTC enumeration**

After staining, whole-slide images were acquired using an Akoya Biosciences PhenoImager at 40x magnification (0.25 um per pixel resolution) in DAPI, AF488, AF555, AF594, and AF647, and autofluorescence channels. Exposure times were guided by prior healthy donors, spiked cells, and kept consistent between samples. Lower exposure scans were used as needed to avoid saturation. These 8-bit images quantified signal in each channel from 0-255. Where necessary, spectral unmixing was applied utilizing the Akoya InForm software. The images were subsequently exported and processed using a HALO image analysis platform. The initial analysis of the entire slide was performed to identify individual cells using nuclear staining and the levels of fluorescence signal in each channel. Adhering to empirically set inclusion and exclusion parameters, customized digital image processing-based segmentation algorithms marked a subset of these cells as possible CTCs requiring further manual verification. These cells were then individually analyzed, considering overall morphology, size, and staining intensity. Following independent analysis by at least two researchers, CTCs were scored. A similar segmentation algorithm was used to mask all CTCs, including nuclei and the entirety of the cytoplasm. The intensity of TROP2 and/or HER2, averaged across this mask and normalized for exposure time, was compared to cut-offs to classify CTCs within specific expression classes. All counts were normalized to 20mL of sample volume.

**CTC Epitope Analysis**

As CTCs vary widely in size, morphology, and intensity, a standardized method was developed to organize individual CTCs across a spectrum of HER2 and TROP2 expression into a series of classes, referred to as ‘Null,’ ‘Low,’ ‘Medium,’ and ‘High.’ To ensure these classes were not only consistent from sample to sample but also represented broadly generalizable levels of expression, various cell lines with known expression levels were processed, stained, and analyzed in an identical manner. These included internally established cancer cell lines (BRx-142, breast cancer; Mel-167, melanoma), cultured in RPMI-1640 supplemented with B-27, epidermal growth factor (20ng/mL), fibroblast growth factor (20ng/mL), and antibiotic-antimycotic and incubated in 5% $CO_2$ and 4% $O_2$, and breast cancer cell lines MDA-MB-231 (RRID:CVCL_0062) and AU565 (RRID:CVCL_1074), sourced from ATCC and cultured as instructed by ATCC. Cut-offs for ‘Low,’ ‘Medium,’ and ‘High’ were based on knowledge of expression levels of these lines, i.e., BRx-142, a CTC line, expresses more TROP2 than most breast cancer lines, while Mel-167 expresses none of the markers and was used as a lower threshold for both TROP2 and HER2. The expression levels in cell lines (BRx142, AU565, MDA-MB-231, and MEL 167) were normalized by exposure and divided into quartiles, categorizing them as low, medium, or high expression. Patient CTCs were then classified according to these thresholds. For tissue specimens, tumor regions of the tissue biopsies were identified by a pathologist. Using a similar quantification approach as was used for CTCs, tumor cells were segmented and phenotyped using HALO software. Cells were identified with DAPI nuclear stain, and the intensity of markers within the nuclear and cytoplasmic compartments for each cell was exported to a data table for statistical analysis. These cut-offs, noted as lines overlaid on violin plots for each respective panel, are shown for reference **(Supplementary Figure 2)**.

**Data availability statement**

All images and data will be made available upon request at Open Science Framework (OSF).

## Authors' Disclosures

Massachusetts General Hospital has been granted patent protection for the inertial separation array and inertial focusing microfluidic technologies used for CTC isolation. M.T., D.A.H., S.M., and D.T.T. are co-founders of TellBio, a biotechnology company commercializing the CTC-iChip technology. All authors' interests were reviewed and managed by Massachusetts General Hospital and Mass General Brigham in accordance with their conflict-of-interest policies.

## Acknowledgments

We are grateful to the patients who donated blood to enable this work. This study was supported by NIH grant K25HL169816 (A.M.); NIH grant RO1CA129933 (D.A.H.), NIH grant U01CA214297 (M.T., D.A.H., S.M.), NIH grant R21CA260989 (M.T.), and NIH grant R01CA255602 (M.T., D.A.H., S.M.), the Howard Hughes Medical Institute (D.A.H.), the Breast Cancer Research Foundation (S.M.), and the National Foundation for Cancer Research (D.A.H.).

## Authors' Contributions

A.M., A.B., D.A.H., S.M., and M.T. developed the concept. A.M., A.B., D.A.H., S.M., Q.E.C., and M.T. designed experiments. A.B., D.A.H., S.M., and M.T. supervised the project. A.M., L.T.N., M.S., Q.E.C., V.R.P., A.D., E.S., K.A.G., K.K., R.B., J.K., K.H.X., and J.W. conducted experiments and analyzed data. R.A., J.F.E., J.W., L.E., C.S.D., S.H., and D.T.T. provided clinical research samples or reagents and gave technical support. A.M., Q.E.C., A.B., R.A., D.A.H., and S.M. wrote the manuscript. A.M., R.A., and Q.E.C. contributed equally to this work.

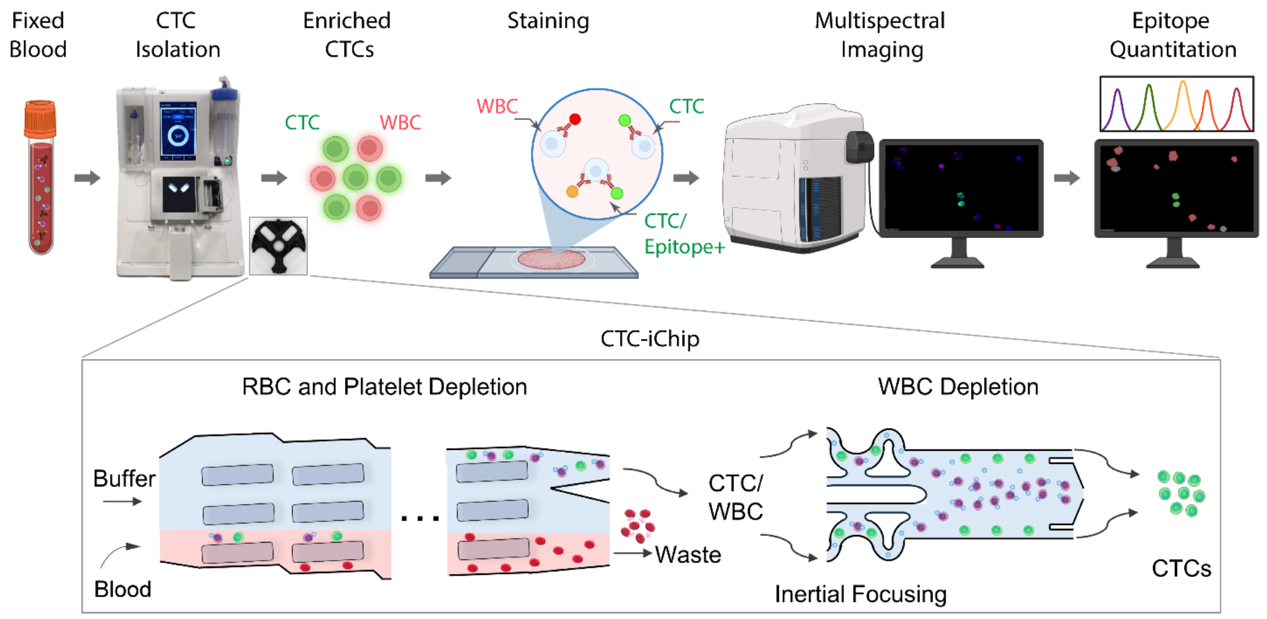

**Figure 1**: **Schema showing the scoring of antibody-drug conjugate (ADC) target expression in Circulating Tumor Cells (CTCs).** CTCs from patients with metastatic breast cancer are enriched from 20 mL blood samples collected and fixed using Streck tubes, followed by processing through the microfluidic CTC-iChip for depletion of hematopoietic blood cells (29,30). Isolated CTCs with residual White Blood Cells (WBCs) are cytospun onto a glass slide, stained with fluorescent antibodies against ADC epitopes of interest, together with shared epithelial/lineage markers (positive identification of CTCs) and WBC markers (negative markers), followed by whole slide imaging (40X) using a multispectral imaging system. For imaging analysis, tumor cells are identified as DAPI+, EpCAM/pan-CK/CK19+, and CD45/66b/16-, and these CTCs are then interrogated for TROP2 and HER2 expression using digital image processing. The inset image shows a schematic diagram of the CTC-iChip system. Red Blood Cells (RBCs), platelets, and plasma are removed using an inertial separation array device, followed by the removal of magnetically tagged WBCs using a magnetic sorter. Nucleated cells are inertially ordered into a single row before flowing into the magnetic sorting channel to provide high-efficiency single-cell sorting, discarding antibody-bound WBCs and collecting unlabeled CTCs.

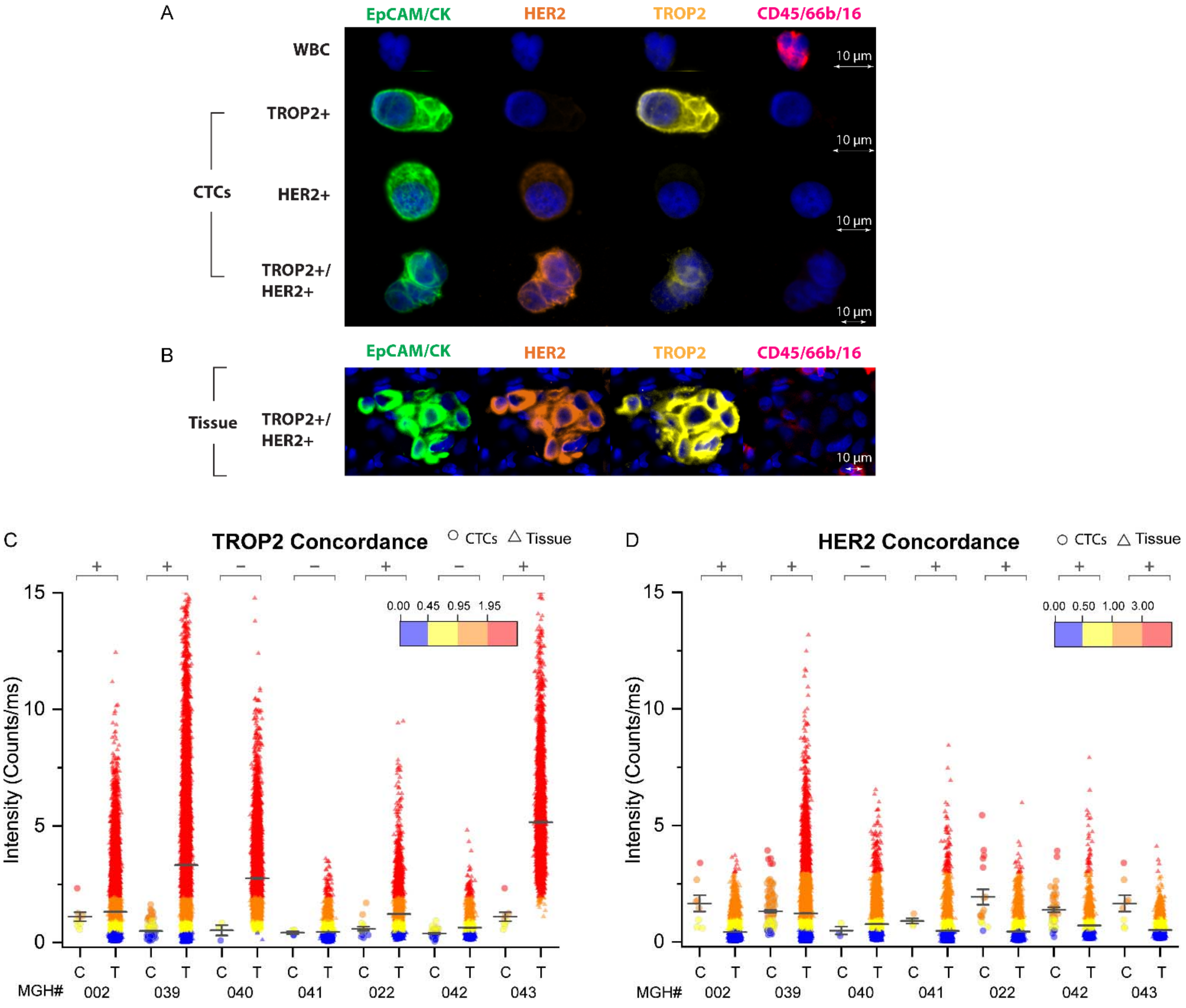

**Figure 2: Comparison of TROP2 and HER2 expression in CTCs and matched tissue biopsies. (A)** Representative images of CTCs isolated from peripheral blood samples of patients with metastatic breast cancer. CTCs staining for the nuclear marker DAPI (blue), and the general tumor/epithelial lineage markers (EpCAM, pan-CK, and CK19 (Alexa Fluor 488; green)), as well as TROP2 (Alexa Fluor 555; yellow) and HER2 (Alexa Fluor 594; orange) are shown. A WBC staining for CD45, CD66b, and CD16 (Alexa Fluor 647; red) is shown as a negative control. **(B)** Immunofluorescence staining of a metastatic tumor biopsy stained using the same markers. **(C)** Comparison of single-cell TROP2 expression in metastatic tumor biopsies versus CTCs in seven matched cases. Staining was performed using the same fluorescent-tagged antibodies. CTCs and single tumor cells in biopsy samples are classified as null (blue), low (yellow), medium (orange), and high (red) for TROP2 expression, using thresholds and expression range established with stable cultured cell lines (**Supplementary Figure 1**).

All tissue samples express some tumor cells with either low, medium, and high TROP2 and HER2 intensities. Concordance (+) is noted between CTCs and tumor biopsy samples if CTCs express overlapping medium or high TROP2 or HER2 intensities. The lack of concordance (-) is noted when CTCs only display low or null TROP2 or HER2 expression despite the presence of medium or high expression in some cells within tumor biopsies. For each matched case, the total number of single cells scored are as follows: MGH002: (biopsy: 24,356; CTCs: 8), 039: (biopsy: 14,720; CTCs: 166), 040: (biopsy: 11,704; CTCs: 3), 041: (biopsy: 5,637; CTCs: 4), 022: (biopsy: 5,061; CTCs; 18), 042: (biopsy: 2,163; CTCs: 49), and 043: (biopsy: 5,617; CTCs: 8). **(D)** Comparison of single-cell HER2 expression in matched tumor biopsies and CTCs, as described above. CTCs and tumor cells in tissue samples were classified as null, low, medium, and high in HER2 expression using thresholds established with cultured cell lines (**Supplementary Figure 2**). Concordance between CTC and tumor biopsies was scored as above.

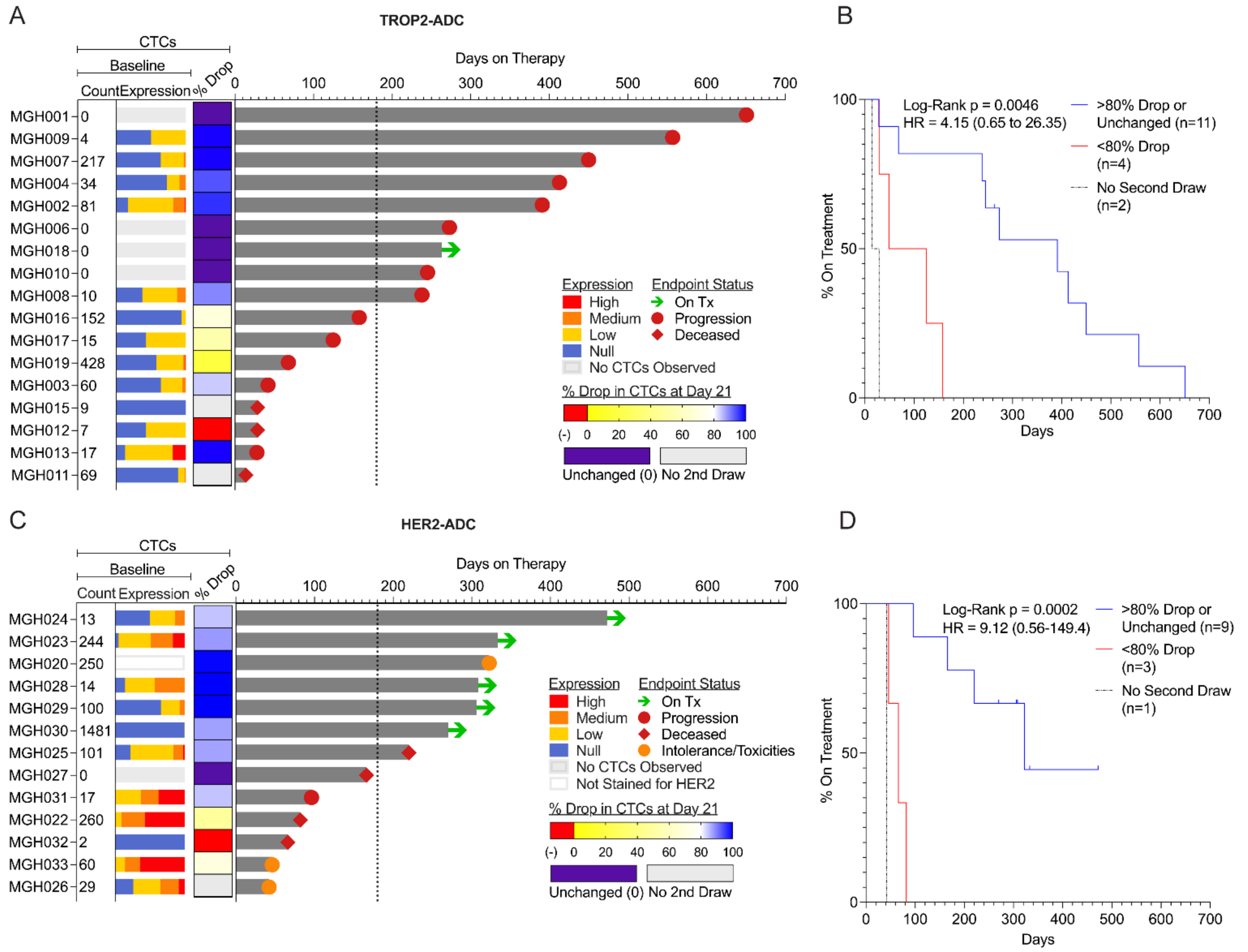

**Figure 3: Clinical response and CTCs in TROP2- and HER2-ADC treated patients. (A)** Analysis of 17 patients with metastatic breast cancer treated with TROP2-targeting ADCs. From left: Number of CTCs isolated from blood (count) at pretreatment baseline, normalized to 20mL; Colored stacked bar graph showing the fraction of CTCs expressing low, medium, and high levels of TROP2 at baseline for each patient (color code shown at right); Percentage drop in CTC counts at the first post-treatment visit (approximately day 21) for each patient (color code shown at right); Swimmer plot showing the duration of TROP2-ADC therapy (days) as a measure of continued treatment efficacy (Time to Progression), with the reason for treatment discontinuation (progression or deceased) noted. The hashed line represents the six-month distinction between strong and poor responses. **(B)** Kaplan Meier plot showing time to progression on TROP2-ADC therapy in patients who either have no detectable CTCs or a greater than 80% decline in CTC counts after three weeks of therapy initiation (blue) compared with patients with a lesser decline or an increase in CTC counts at three weeks (red). Log-Rank P-value and hazard ratio (HR) compare '>80%' and '<80%' groups. Patients from whom no on-treatment blood draw was available are shown (dashed line) but are not included in statistical calculations. Evaluable patients had clinical or radiographic assessment and pre and on-therapy blood draws. Censored patients who remain on treatment are noted as hash marks. **(C)** CTC characteristics and Swimmer Plot analysis of 13 patients with metastatic breast cancer treated with HER2-targeting ADC. Features are as shown for panel (A). **(D)** Kaplan Meier plot showing time to progression on HER2-ADC therapy as a function of CTC counts at 21 days. Features are as described in panel (B).

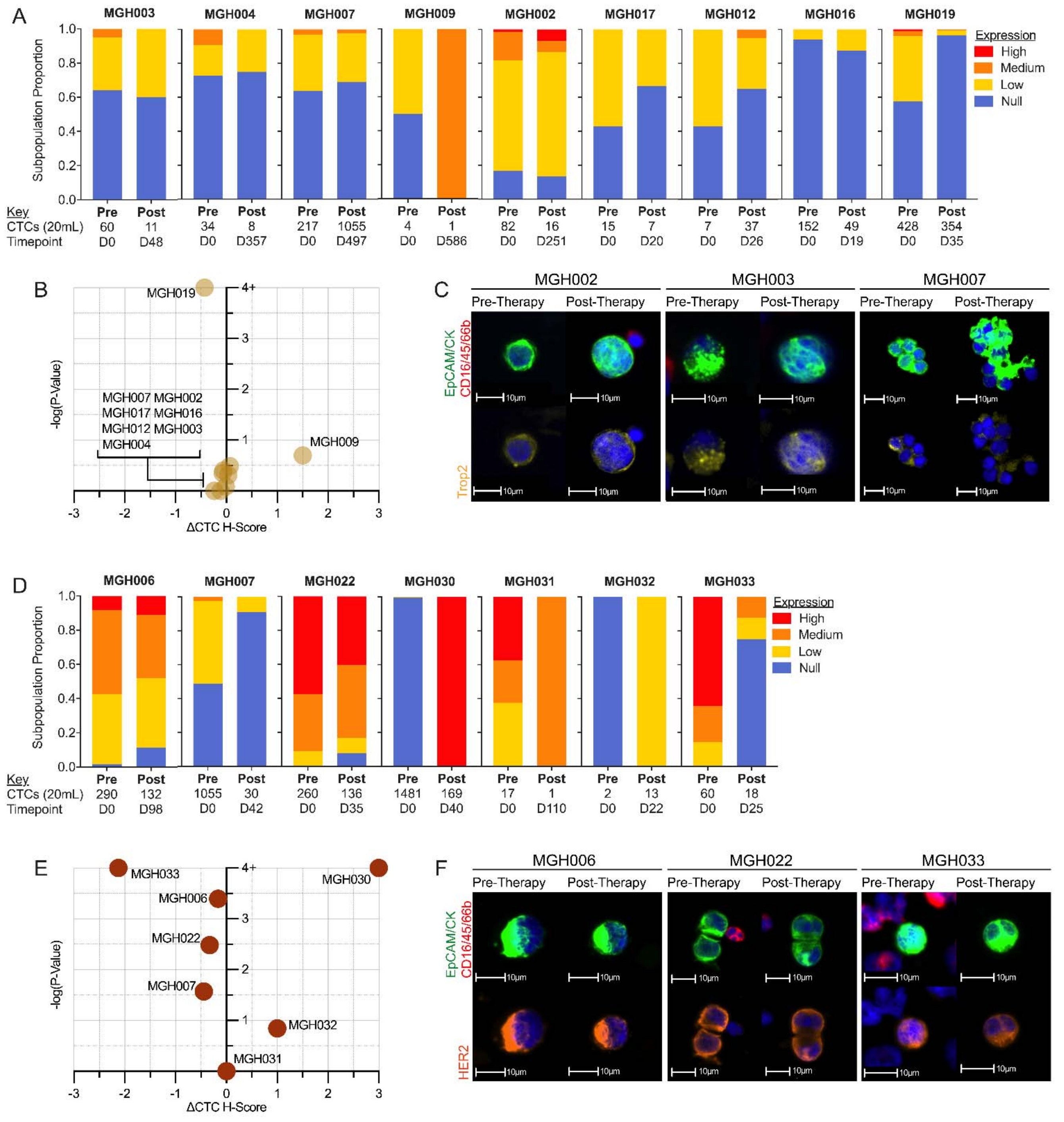

**Figure 4: Persistence of TROP2 and HER2 epitope expression in CTCs at the time of progression on TROP2- and HER2-ADC. (A)** Bar graphs show the distribution of TROP2 expression in CTCs collected from nine patients, comparing pretreatment initiation (left) with matched samples at the time of disease progression and termination of therapy. The number of CTCs (normalized to 20mL) and the timepoint of each draw relative to treatment initiation are noted below the plots. **(B)** Plot showing both the change in TROP2 CTC H-score from baseline to recurrence as well as the log(P-value) (Fisher's exact test) for each of the nine patients on TROP2-ADC, quantifying the significance of changes in epitope expression between matched pretreatment and ADC resistant CTCs. H-score is defined as 1×proportion of 'Low' cells + 2×proportion of 'Medium' cells + 3×proportion of 'High' cells. Only one out of nine patients (MGH009) shows a meaningful (>1) difference in H-score between draws (increased epitope expression at recurrence). **(C)** Representative images of TROP2 expression in matched CTCs from pre-therapy and recurrence samples, demonstrating unchanged cytological features, including cell morphology and CTC cluster formation. **(D)** Bar graphs showing the distribution of HER2 expression in CTCs collected from seven patients prior to initiation of treatment and at disease progression. CTC number (normalized to 20mL) and time points of each draw relative to treatment initiation are noted below the plots. **(E)** Plot showing both the change in HER2 CTC H-score from baseline to recurrence and the log(P-value) (Fisher's exact test) for each of the seven patients. One patient showed a statistically significant decline in HER2 expression, and one patient showed increased HER2 at progression, with the remaining five patients showing no change in CTC expression. **(F)** Representative images showing similar HER2 expression and CTC morphology in matched pre-therapy and recurrence samples.

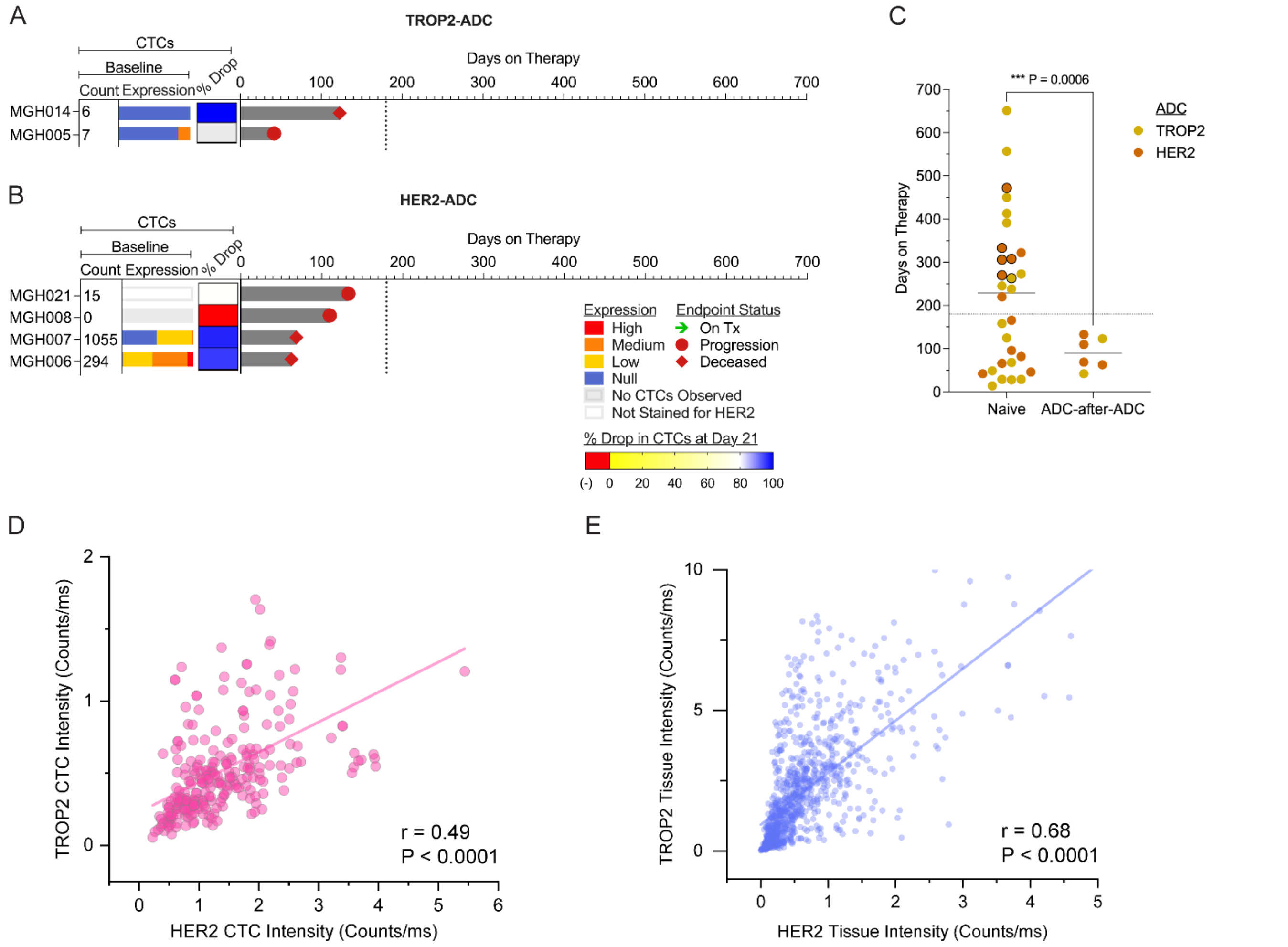

**Figure 5: Poor second-line response in patients receiving sequential ADCs. (A-B)** Analysis of two patients with metastatic breast cancer treated with TROP2-targeting ADC after having progressed on a HER2-targeting ADC (A) and four patients treated with HER2-targeting ADC after having progressed on a TROP2-targeting ADC (B). From left: Number of CTCs (count) isolated at pretreatment baseline (normalized to 20mL); Colored stacked bar graph showing the fraction of CTCs expressing low, medium, and high amounts of TROP2 or HER2 (respectively) at baseline for each patient (color codes at right); Percent drop in CTC counts at the first post-treatment visit (approximately day 21) (color code at right); Swimmer plot showing the duration of second-line therapy (days) as a measure of continued treatment efficacy, with the reason for treatment discontinuation (progression or deceased) noted. The vertical dashed line denotes the 6-month cut-off for a strong versus poor response to the second-line ADC. **(C)** Scatter plot showing time to progression (days) in six patients receiving second-line ADC therapy ("ADC after ADC"; HER2 followed by TROP2 (n=2) or TROP2 followed by HER2 (n=4)) versus patients receiving first-line ADC therapy (TROP2 (n=17) or HER2 (n=13)). ADC color code describes either the first-line ADC or second ADC used in the "ADC after ADC" cohort. Data is separated by cohort, and darkly circled points represent patients who remain on treatment. A hashed line represents the 6-month cut-off for strong versus poor ADC response, and two solid lines indicate the median time on therapy for each group (first-line: 229 days vs second-line: 90 days, P = 0.0006 by Welch's T-test. **(D-E)** Correlation between TROP2 and HER2 expression within single CTCs (D) and single cancer cells from tumor biopsies (E), pooled from seven ADC naïve cases of metastatic breast cancer, showing moderate coexpression of these two ADC epitopes within single breast cancer cells.
